# Perceptions on the collection of body fluids for research on persistence of Ebola virus: A qualitative study

**DOI:** 10.1371/journal.pntd.0008327

**Published:** 2020-05-14

**Authors:** Ruth Kutalek, Florence Baingana, Stephen Sevalie, Nathalie Broutet, Anna Thorson

**Affiliations:** 1 Unit Medical Anthropology and Global Health, Department of Social and Preventive Medicine, Center for Public Health, Medical University of Vienna, Austria; 2 WHO country office Sierra Leone, Freetown, Sierra Leone; 3 UNDP-UNFPA-UNICEF-WHO-World Bank Special Programme of Research, Development and Research Training in Human Reproduction (HRP), World Health Organization, Geneva, Switzerland; Faculty of Science, Ain Shams University (ASU), EGYPT

## Abstract

**Background:**

Against the background of the international public health emergency related to the Ebola outbreak in the Democratic Republic of Congo, in addition to other recent large Ebola epidemics, the issue of transmission due to viral persistence from survivors’ body fluids is becoming increasingly urgent. Clinical research in which body fluids play a role is critical and semen testing programs are part of the suggested response to the outbreak. Broad acceptance and understanding of testing programs and research, often in resource poor settings, is essential for the success and sustainability of clinical studies and an accurate epidemic response. Study participants’ perceptions on the collection of body fluids are therefore relevant for the programmatic planning and implementation of clinical studies.

**Study aim and methods:**

In this qualitative study we aimed to explore the perceptions on bio-sampling in the Sierra Leone Ebola Virus Persistence Study (SLEVP study). We were interested to understand how norms on gender and sexuality related to perceptions and experiences of study participants and staff, specifically, in what way perceptions of the body, on intimacy and on body fluids related to the study process. We purposively sampled former study participants for in-depth interviews and focus-group discussions. We conducted 56 in-depth interviews and eight focus group discussions with 93 participants. In a participatory approach we included study participants in the analysis of data.

**Results:**

Overall the SLEVP study was well perceived by study participants and study staff. Study participants conceived the testing of their body fluids positively and saw it as a useful means to know their status. However, some study participants were ambivalent and sometimes reluctant towards sampling of certain body fluids (especially semen, blood and vaginal fluid) due to religious or cultural reasons. Self-sampling was described by study participants as a highly unusual phenomenon. Several narratives were related to the loss of body fluids (especially semen) that would make men weak and powerless, or women dizzy and sick (especially blood). Some rumors indicated mistrust related to study aims that may have been expressions of broader societal challenges and historical circumstances. These reservations could eventually be overcome by guaranteeing confidentiality and privacy and by comprehensive professional counseling.

**Conclusion:**

In the course of the sampling exercise, study participants were often obliged to transgress cultural and intimate boundaries. It is therefore important to understand the potential importance some of these perceptions have on the recruitment of study participants and the acceptability of studies, on a symbolic as well as a structural level. In order to capture any reservations it is necessary to provide plenty of possibilities of information sharing and follow-up of continuous consent.

## Author summary

Recent studies have shown that the Ebola Virus might persist in body fluids of survivors of the disease. Clinical research in which body fluids (semen, vaginal fluids, blood, sweat, tears, breast milk and rectal fluids) are sampled play an increasingly important role, specifically in light of growing EVD epidemics. The success of these studies, e.g. how many participants are recruited in a study and how many are staying until the end of a study, is highly dependent on the participants’ cooperation and understanding of testing programs. However, until now there has been only little research on how studies and testing programs in which body fluids are sampled are perceived and understood by study participants. In this study we were therefore interested to understand how study participants perceived the sampling and collection of body fluids and how their cultural or religious background may influence the willingness to participate and stay in a clinical study. We conducted one-to-one interviews and focus-group discussions with 93 former study participants of a viral persistence study. We found that overall study participants conceived the testing of their body fluids positively and saw it as a useful means to know their status. However, some study participants were ambivalent and sometimes reluctant towards sampling of certain body fluids (especially semen, blood and vaginal fluid) due to religious or cultural reasons. Self-sampling was described by study participants as a highly unusual phenomenon. Participants explained that the collection of certain body fluids would make men weak and powerless (especially the sampling of semen), or women dizzy and sick (especially blood). Rumors indicated that some participants mistrusted the study aims. In the course of the sampling exercise, study participants often felt that they had to transgress cultural and intimate boundaries. We conclude that it is important to understand the potential importance some of these perceptions have on the recruitment of study participants and the acceptability of studies. The understanding of the socio-cultural context of clinical research is relevant for the programmatic planning of such research.

## Introduction

Sierra Leone was one of the countries most heavily impacted by the West African Ebola Virus Disease epidemic which saw an unprecedented number of survivors. By March 2016, when the WHO Director-General declared the end of the Public Health Emergency of International Concern, there had been 14,124 confirmed, probable and suspected cases and 3,956 deaths [1].

During and after the outbreak there have been several studies on the persistence of Ebola Virus (EBOV) in body fluids, especially in semen [2–4] suggesting sexual transmission of the virus from male survivors [5,6] or other forms of viral persistence-derived transmission [7–9].

In studies on viral persistence body fluids are sampled from male and female EVD survivors, often in resource poor settings. Broad acceptance and understanding of testing programs and research is essential for the success and sustainability of clinical studies, specifically for the enrolment and retention of study participants. It has been shown, for instance, that perceptions of blood sampling significantly influenced study uptake and loss to follow-up [10–12]. In many cultural contexts body fluids such as blood, menstrual blood, semen, urine, feces, or sweat are not “neutral substances” but endowed with meaning [13, 14]. They may be considered as pure or polluted, as powerful substances, inflicting harm or as curative agents. From anthropological research we know that Muslim men may be especially conflicted about delivering semen samples in a clinical setting [15]. For female study participants it is important to understand how notions of purity and shame across different socio-economic, ethnic and religious groups might influence perceptions and acceptability e.g. of vaginal self-sampling [16,17]. These aspects, however, have so far rarely been considered in studies on viral persistence.

As we are faced with a growing number of EVD outbreaks (e.g. the current outbreak in DRC, as of 25 Feb 2020 3444 cases reported [18]) evidence on viral persistence e.g. of Zika virus is also increasing [19]. Clinical research in which body fluids play a role is becoming more important. Study participants’ perceptions on the collection of body fluids will therefore become more relevant for the programmatic planning and implementation of clinical studies.

In this qualitative study we aimed to explore the perceptions on bio-sampling in the Sierra Leone Ebola Virus Persistence Study (SLEVP study). The SLEVP study was established to investigate persistence of Ebola Virus (EBOV) in body fluids (semen, vaginal fluid, menstrual blood, urine, rectal fluid, sweat, tears, saliva, and breast milk when applicable) of 120 male and 120 female EVD survivors, and is described in detail in Deen et al. [20]. Participants received counseling prior to the sampling process, as well as two weeks later when they received their test results, described in detail in Abad et al. [21].

In this process evaluation we wanted to explore perceptions of study participants and study staff in the Sierra Leone Ebola Virus Persistence Study (SLEVP study) regarding the implementation of the study and the specimen process itself. Employing a meaning centered and critical approach, we were interested to understand how norms on gender and sexuality related to perceptions and experiences of study participants and staff, specifically, in what way perceptions of the body, on intimacy and on body fluids related to the study process. Furthermore we sought to explore how experiences of the Ebola epidemic impacted on the implementation of the study as well as on the perception of the sampling process.

## Material and methods

The study was conducted between April and June 2017 at the two SLEVP study sites 34 Military Hospital (MH34) and Lungi Government Hospital (LGH). We invited all four counselors (2 from each site) and 13 purposively selected staff from all professions that were part of the SLEVP study, such as medical doctors, study nurses, lab technicians, cleaning personnel and security personnel for an interview. The selection took place in cooperation with WHO staff who were involved in the SLEVP study.

We purposively sampled former study participants with diverse religious background, age and marital status for in-depth interviews and focus-group discussions. By selecting participants with diverse social characteristic we expected to get a broader spectrum of perceptions regarding our research questions. For each study site four FGDs were conducted, one with older men, one with older women, one with younger men, and one with younger women. We aimed at homogenous groups in terms of gender and age so that group members would feel comfortable to answer our often intimate questions in the group. The FGDs were an important complement to in-depth interviews as some of the participants felt more at ease to talk in groups and/or were encouraged to tell their own perspective after having listened to their colleagues. Former study participants and study staff were informed about the evaluation study by the study receptionists and community liaison persons. Participants already knew them from the SLEVP study and trusted them.

Overall we conducted in-depth interviews and focus group discussions with 93 participants. We conducted in-depth interviews with all four counselors and 13 SLEVP study staff (doctors, nurses, lab technicians, community liaison persons, cleaning and security) (Table 1) and 31 SLEVP study participants. Additionally we conducted eight focus group discussions with 45 SLEVP study participants (Table 2).

**Table 1:** Characteristic of former SLEVP study staff (IDI: in-depth interviews, MH34: 34 Military Hospital; LGH: Lungi Government Hospital).

	N	female	male
**Counselors**	4	3	1
**Study staff (study doctors, study nurses, community liason, lab technicians, cleaning, security**	13	6	7
**N**	**17**	9	8

**Table 2:** Characteristic of former SLEVPS study participants (IDI: in-depth interviews, FGD: focus group discussions).

	N	age range	Muslim	Christian	married	single	widowed
**IDI female**	18	18–75	12	6	8	9	1
**IDI male**	13	21–65	10	3	8	5	
Overall	**31**						
**FGD 1,2 female younger**	12	19–26	7	5	5	7	
**FGD 3,4 female older**	12	31–55	7	5	3	3	6
**FGD 5,6 male younger**	10	20–29	7	3	4	6	
**FGD 7,8 male older**	11	30–62	8	3	8	2	1
FGD N	**45**						
**Overall N**	**76**						

All interviews were carried out by experienced interviewers who were undergraduate university students in social work or social sciences. All interviewers spoke English and Krio, two also spoke Temne. The three male and one female interviewers had formerly been trained in and conducted qualitative health studies in Sierra Leone and were competent in qualitative interviewing. They received an additional two-day training by the study lead (RK) on principles of qualitative research, in-depth interviews, focus-group discussions and medical ethics. The training also entailed the use of the specific interview guidelines and the informed consent. The study lead closely supervised the interviewers in the field and gave them constant professional feedback on interview techniques and non-verbal communication skills.

The interview guides were co-developed by all five authors in a discursive process, guided by the research questions. They were translated into Krio by the interviewers, under close supervision of SS, FB and RK, and extensively discussed. They were then re-translated into English for quality control. The duration of the in-depth interviews was between 30–50 minutes. Focus group discussions lasted about 1.5 to 2 hours each and were carried out by 1–2 interviewers, depending on the availability of interviewers. All male groups were interviewed by male interviewers, the female groups were interviewed by our female interviewer. Most interviews with former study participants were conducted in Krio, some in English and two in Temne. In two of the focus groups one or two participants only spoke Temne; these FGDs were conducted bilingually by the Temne-Krio-speaking interviewers. Some of the fellow participants who were also bilingual supported with the translation. Most interviews with study staff were conducted in English. All interviews were voice-recorded with the consent of the interview partners.

Most transcriptions were conducted by a specifically hired research assistant who also translated the interviews from Krio to English. The Temne interviews were transcribed and translated into English by the Temne speaking interviewer. The other three interviewers transcribed two interviews each. All interviews were checked for completeness and accuracy and imported into the software atlas.ti for qualitative content analysis. Instead of names we used unique ID codes.

### Analysis

In a first phase we read through around one third of the transcribed interviews and assigned preliminary codes that emerged from the data to catch important concepts and categories. Then a very broad analysis was performed that summed up the main themes. With this preliminary analysis in hand we invited all study participants again and asked them on their feedback on these first results. As ownership and confidentiality of study participants is most important, we asked each interview partner how he or she would like to be informed about the preliminary results of the study and most agreed to be called again and informed in groups. At each study site we therefore held three separate dissemination meetings: one for the group of former study staff, one for female participants and one for male participants. We communicated these first results of the study and encouraged participants to discuss them with us. In this participatory approach we wanted to identify the most important themes for the participants, to clarify issues that may have been understood differently and to give participants the opportunity to air any concerns they had. The results of these meetings informed the second phase of analysis of the data and allowed a better definition and refinement of the codes and grouping in categories. Throughout the study ideas about the data were documented in theoretical memos. In an iterative and reflexive process of data immersion we searched for meaning and insightful findings [22].

In line with the analytical emphasis of this paper we identified three major themes: the understanding of the study and the recruitment process, the perceptions on and experiences of intimate sampling (semen, vaginal and rectal fluids) and the perceptions on and experiences of the sampling of blood and other body fluids.

### Ethics statement

The research protocol was approved by the Sierra Leone Ethical Review Board and the WHO Ethical Review Committee. All data produced in the project are strictly confidential. We did not mention any real names, either in the transcriptions or in the publication. Prior to the interview all participants have been informed about the project and its objectives, the purpose of the interviews, and the use of the data for scientific purposes. Interviewers and the research assistant signed a confidentiality agreement.

The informed consent was read to them carefully in their preferred language and after agreeing to it participants and interviewers signed (or thumb marked) the informed consent form. All participants were informed about the voluntary character of this study and guaranteed strict confidentiality. They all consented that the interviews were being voice recorded. Study participants were compensated for their travel costs and time spent during the interview.

## Results

### Understanding the viral persistence study and the recruitment process

Participants related to us how they were first informed of the study through information meetings, where aims and bio-sampling were explained. In the beginning of the recruitment process potential participants were concerned about confidentiality. There were also fears and rumors among survivors that study participants would be infected with EVD or that they would be used as guinea pigs in the study. Moreover there were rumors that the blood drawn in the study would be sold by the government, used during elections or as a treatment in case of another epidemic.

We never wanted to come. We were afraid. We just thought that because they missed to kill us at the [treatment] centers, it was through the test [study] they were going to get rid of us. (…) At first we were afraid, but thereafter we became confident. Onto the end I had no problem. Former SLEVP study participant IDI, male

They said the blood they collected from us was going to be kept and used during elections. Some said they kept the blood perhaps another disease will come so that they could use it to treat the other patients. Those were some of the things they said. Former SLEVP study participant IDI, female

Several participants mentioned rumors they heard on the study that the blood collected would be sold, that they “would make money out of our fluids” or that body fluids would be kept for a future epidemic. Some said that they were afraid that “the white people wanted to use us” and “sell our lives”. Not all believed in these rumors but clearly there was quite some anxiety among the participants initially. Study staff related that they employed different strategies to overcome those concerns: they talked to the survivors, the community elders and other representatives and tried to convince them on the good intentions of the study and that they would themselves profit from knowing their status. Moreover, the very fact that several study participants knew the staff involved in the study from the EVD Treatment Unit (ETU) in which they were treated opened many doors. Study staff also mentioned that the very fact that survivors were employed as liaison officers was a trust building measure.

Staff as well as participants highly appreciated the confidentiality and privacy of the project which was realized at different levels. Participants received a unique ID number and were addressed only with this number instead of their names. On several occasions participants related to this measure as trust building. The study sites were separated from the main clinic buildings and according to our interview partners no one except study staff and participants had access. Moreover, care was taken not to disclose persons as participants in front of family or community. Several participants and staff mentioned that when they met accidentally outside the study compound they would not take notice of each other, or when participants were called and did not answer the call personally the reason for the call would not be disclosed. Furthermore, informing the public about the project, especially the detailed procedures of the project, e.g. what kind of samples were provided and in what way, was kept to a minimum; more information was only provided at the meetings where the recruitment took place and details were disclosed in the individual counseling sessions. Participants often stressed that the study “was a secret” or “like a secret society”. Many participants related that little was known about the study outside the survivor community, because they were asked to “keep the information to themselves”.

They told us earlier that we should not disclose any information to the community people and there is no need for us to explain to them that we are engaged in dry jack [masturbation], given out semen, and body water [body fluids] for test to be conducted. We felt the entire process should be a secret that we should not disclose to them. Former SLEVP study participant FGD, male group

They told me that those of us registered for the study must not tell other people about it. When I returned home from the study, I was always with my children and I explained nothing to them. Former SLEVP study participant IDI, female

Participants greatly appreciated the friendly and cordial atmosphere of the project and that staff treated them in a kind and respectful manner. They also appreciated the free medical care and the reimbursements they received during the project.

### Sampling of body fluids

From our interviews it became clear that counselors and nurses, but also lab technicians, doctors and liaison persons were important to inform the participants on the sampling of body fluids. For several reasons participants were sometimes reluctant or not able to provide a sample (the most problematic were semen, vaginal and blood samples) and staff negotiated their cooperation. Study staff were available to assist participants with providing samples and doing the self-swabs, especially when the participant did so for the first time. Inside the sampling tent posters explained the detailed procedures for each sample in pictographs. Our interview partners related that at the beginning of the study, staff would help with the sampling of vaginal and rectal fluid, saliva, tears, sweat and urine. At subsequent visits participants were more confident and often did the sampling without help. For providing the semen sample all staff would leave the tent after having instructed the male participant.

Specifically for the intimate sampling, privacy and confidentiality were of outmost importance. Overall study staff thought that most participants were fine with the sample taking and that participants were rather concerned about their health and knowing their status than with personal feelings of shame and fear. Most participants agreed on the principal necessity to test body fluids. However, there were certain sampling procedures of body fluids, such as the sampling of semen through masturbation or the sampling of vaginal fluids during menstruation, many participants felt very strongly about. They were unusual for most of the participants and initially led to fear, shame, reluctance and open opposition of some.

### Male intimate sampling

Almost all the study staff acknowledged that the sampling procedure for semen was a challenge for most male participants. Staff mentioned several reasons why participants were not able to produce a semen sample. Some argued that it was because of religious reasons, some said that it was due to the sickness that made them impotent. Others argued that participants related to them that they would need their wives in the process. Staff occasionally also mentioned that contrary to what they had been advised, participants would often not abstain from sex before they were scheduled to come for the sampling.

Majority of the things went well except for the men, some will come and give all other body sample but when it comes to the semen, they will give an excuse that they are religious and don’t do such thing, especially the Muslims a lot of them were refusing to give a sample even when you booked an appointment with them. Former SLEVP study staff, female

… some participants even when we advised them to abstain three days before sample collection they won’t adhere to that advice. They would have sexual intercourse the night before sample collection. When they came for the sample collection they won’t be able to give the sample. Former SLEVP study staff, female

For the participants religion was considered an important issue for the provision of semen samples in several aspects. Some men were highly uncomfortable to masturbate and considered it indecent and against their religion. Furthermore, especially during Ramadan, sexual intercourse and more so masturbation is considered haram (religiously forbidden). While many participants thought that the pornographic film greatly helped them during masturbation, some said they would not have needed it or that they didn’t want it for religious reasons. Some Muslim men openly objected to having pornographic movies shown.

Just as my brother has said, it [masturbation] is understandable in the urban centers a bit, but in the rural areas it is highly prohibited. In the village, they refer to anybody who does that as unreligious. Former SLEVP study participant FGD, male group

We were not feeling fine because God does not permit us to take out [semen], without a woman, it is not decent at all, and we are just doing it because of our health and our status. Everything we were doing, we say it is confidential but it was painful. Former SLEVP study participant FGD, male group

Even though Muslims generally do not approve of masturbation, several staff and participants argued that it would be tolerated if it were done for a medical reason.

Normally especially the elderly people and the Muslims were not comfortable with it. Some people were saying it is forbidden but we had meetings with the Islamic council before we started the study. We asked them what Islam will tolerate. They told us that yes during Ramadan Islam will not tolerate such. But if it is not in Ramadan and it’s for your well being you are permitted to do it. And that was settled. Former SLEVP study staff, female

For me, as long as it is for my health, (…) I just think God can understand because it is not something intentional, we were doing it for our health. To our people it is really something bad. Former SLEVP study participant FGD, male group

Participants often related that they are “not used” to masturbate and on several occasions they talked about loosing power or even becoming sick through masturbation, because they perceived masturbation as fundamentally different to having sexual intercourse. They said that in their communities people would not talk about it openly and they would consider it a sin. Three persons also explicitly mentioned that persons engaging in manual ejaculation are considered homosexuals by their communities.

For example, when we come and give the semen I will have to buy some drinks when I get home to replace what I have lost, that was how I used the money. Former SLEVP study participant IDI, male

We were just doing but it weakened your system because when you jack your penis [masturbate] … when you engage in sex with a woman you feel relaxed on top of the lady the penis is controllable but when you ejaculate the penis gets weak. Former SLEVP study participant FGD, male group

People who do masturbation are perceived badly by the community people, they will always class such person as unserious and being homosexual. Former SLEVP study staff, male

Reservations of participants towards providing semen samples on some occasions also led to open resistance. Staff argued that the withholding of semen samples was in some instances done deliberately because this did not only happen in the beginning of the study but also later, when participants were already accustomed to the procedures. They related that some patients realized they would still get reimbursed even when no sample was provided. In the view of staff resistance took different, more or less subtle forms, from the understandable rejection to give a sample e.g. due to religious reasons, to a coordinated collective form of resistance.

… some of them would say I would give blood but would not give the semen. Former SLEVP study staff, male

Some followed their colleague who refused to give sample while others who were hesitating also joined their colleagues (…). In such cases when we noticed that some were only coming for the cash and not to give sample, we started withholding their cash and demanded they give the sample first because if such continues we cannot get the actual outcome of the research. With these methods we were able to solve some of the challenges. Former SLEVP study staff, female

Some male participants who were either not able or not willing to produce a semen sample were suspected to put gel or similar looking substances into the test tubes or bring a semen sample from outside. However, participants never said that they deliberately deceived study staff, but they knew that at least at the beginning of the study there would be no consequences when they were not able to produce a semen sample. This strategy, as also mentioned in the previous citation, was later changed.

(…) if you are not careful they bring the semen from home because they said unless there is a woman they can’t produce semen. Former SLEVP study staff, male

We understood that some men because they were not ejaculating they were ashamed to come out without the semen sample. So, we speculated that they were putting the hand sanitizer in the test tube. Former SLEVP study staff, female

If you could not produce for this week, they would encourage you; perhaps you could produce in another week. They gave the same transport refund even if you could not produce, or even if you produce only one fluid they would appreciate. Former SLEVP study participant FGD, male group

### Female intimate sampling

Compared to the male participants, staff considered female participants to be unproblematic and cooperative in terms of intimate sampling (vaginal and rectal swabs). When relating to sampling in general, staff would usually highlight the challenges they had with male participants to collect semen. Some staff acknowledged that there was initial fear and shyness but overall staff were more occupied with male concerns, even though strong reservations towards intimate sampling were present in both sexes.

For the women there was no problem, they were so cooperative. Former SLEVP study staff, male

For the eye water and breast milk there was no problem except for the vaginal fluid. Especially, for the aged women, they were a little bit shy. (…) They agreed when they understood the procedure. Initially, they thought that there will be a man present. Also we told them that they could even do it alone, and they would only be assisted by a nurse if they need it. We were not having any problems with the women. Former SLEVP study staff, female

Some of them were afraid except when I explained to them that is so tiny like the feather of a chicken and this gave them confidence. (…) it was a mixed reaction, some accepted it whilst others rejected. Former SLEVP study staff, female

However, several female participants described their concerns quite differently. Many did not express their reluctance with sampling procedures openly but rather talked of having experienced fear, shame and embarrassment. One woman related that she did not want to disappoint the elders. Others were quite frank and described their struggle with the staff.

After the whole process was explained to us and what they will be taking from us I really felt bad and I didn’t want to reject my elders that’s why I participated in the study. And until it finished I did not encounter any problem. Former SLEVP study participant IDI, female

The first time I came on this study I did not feel good especially the woman side. To be honest with you I was so much adamant with the nurse, but she encouraged me and even removed her pant for me as a way to give me more courage. I gave them the different samples they needed from me. Also the second time again when I came I was still adamant to give the samples. They still counseled me and I gave them the sample. The last time there was no problem between us because I realized that they were trying to help me know my status. Former SLEVP study participant FGD, female group

Some study participants were specifically concerned about the inclusion of elderly ladies into the study.

For instance a young nurse ordering an old woman to remove her pant is somehow embarrassing. Former SLEVP study participant FGD, female group

Some women considered the collection of menstrual fluids specifically difficult. Menstrual blood is considered unclean and participants on several occasions indicated that they were concerned what was done with the sample. One important strategy to overcome shame and embarrassment of the participants was to counsel and talk to the female participants, to treat them in a respectful way and by showing one’s own vulnerability and nakedness.

It was only for the collection of menstrual fluid I know that it was not fine because some women considered it as unclean. Former SLEVP study staff, female

Part [A] As for me what I hate in the whole study process is the vaginal swabbing especially the menstruation. I always ask myself what they are going to do (with) it. (…)

Part [B]: As my colleague said menstruation and the blood is the problem. We really want to know the reason for taken them. Because women find it very difficult to give it out especially to foreign people you don’t know. Former SLEVP study participant FGD, female group

To do the menstruation test was the greatest difficulty I faced in the room. The nurse asked me to remove my pant in order to do the test I told her that I am ashamed to do it. Fortunately, for me the nurse also was on her menstruation period so as a result she removed her pant and shows me how to do the test. Former SLEVP study participant FGD, female group

Many women, especially the younger generation, had no problem whatsoever, either with the collection of menstrual blood or with the vaginal swabbing in general. At the second or third appointment most of them also got used to the procedures and did not need assistance from the nurses any more.

On the first day somebody did it for me and the second the third and forth I did it for myself. Former SLEVP study participant IDI, female

When we come they will tell us not to me ashamed, they are female and am also a female so let me not be ashamed, so whatever they asked me to, let me do. They talk to us fine and encourage us, and we do what they told us, they gave us hot water to drink for us to sweat, so all that. Former SLEVP study participant IDI, female

### The sampling of blood and other body fluids

Female and male participants likewise frequently mentioned the blood draw as a highly uncomfortable event, the experience of which clearly went beyond the mere pain–the drawing of blood was often related to the loss of strength and power. Participants not only mentioned the frequency of the blood draw but also the amount that they often considered “too much”. Female participants were specifically concerned about the “blood loss” that in their perception would cause headache and dizziness. Some male participants also felt that the blood draw would influence the semen sampling.

The only thing as I told you was the too much blood they removed from me. That gave me headache. They said if they did not remove enough blood from people they would not do the sample test, so they removed enough blood from people. That was the only problem I had with them. Former SLEVP study participant IDI, female

When they remove their blood some men complain of shortage of blood which makes their heads spin it [and makes] dry jack [difficult]. We keep on telling them that the 1ml blood does not do anything to their health. Former SLEVP study staff, female

Participants often narrated that they had to replace the blood that was drawn with other substances such as ORS, “blood tonic”, “blood syrup” or some special drinks that are considered blood building.

There was a time I was seriously affected after donating my blood. There was a time I donated blood I felt a pain in such a way that I had to buy blood syrup. As for me, I drank raw eggs for three days before I came to normal, because I felt dizzy when I donated Former SLEVP study participant FGD, male group

What we do not want is the removal of blood for test. If they come again and say blood test we will not take part. All of them mean the same. The blood test made us dizzy. Former SLEVP study participant FGD, female group

Staff also mentioned that even though participants had been informed of and had agreed to the blood draw (e.g. by signing the informed consent), when it came to the actual procedure many participants were reluctant and even resisted to undergo it. Some were quite firm in their opinion that if they were invited for another study they would not join if regular blood draws were a part of it.

They really do give us tough time. They lament to us after they have gone through and agreed to all what they have been told; when they reach to us to collect the specimen especially the blood then they start to grumble and say had I known I would not have come. Former SLEVP study staff, male

They were afraid, they thought we were going to draw huge amount of blood from them except when we told them that we were going to take 5 ml of blood and that it would not exceed. We also show them the sample and they accepted it. Former SLEVP study staff, female

Staff and participants rarely mentioned challenges in collecting or self-swabbing of other body fluids. Only the sampling of tears was described as difficult to some of the staff and the participants. Uneasiness about the rectal swab was explicitly mentioned by three participants and one staff. Several participants were highly concerned where their samples would be taken. Most of these anxieties related to venous blood but three participants also mentioned other body fluids such as menstrual blood and semen.

What I want to say is about the blood collection. It is number one because every time we came was blood, every time we came was blood. Some of us were afraid because we did not know where our blood was taken to. (…) We were getting rumors that a ship was taking our blood away. For some of us we prayed that wherever our blood was taken to with negative intent let our blood be dark. Former SLEVP study participant FGD, female group

I was thinking before when they came and collect our sperm, what are they doing with it? That what I was thinking. Where are they going with them? Because we are knowing the result but where are they going with them? Former SLEVP study participant IDI, male

## Discussion

This is the first study that analyzes perceptions on the collection of bio-samples in medical research on Ebola virus disease. It shows how important it is to understand broader socio-cultural contexts in which medical research is taking place. Perceptions of the body and on sexuality are socially and culturally constructed. They are deeply influenced by historical realities, by ethnic affiliation, power relations, gender roles, concepts of morality, education and religion. Moreover, medical studies that take place in or shortly after epidemics have to consider the specific governance dynamics of the response (or post-response efforts), as well as psychosocial implications of (post-)emergencies.

A study investigating Ebola viral persistence in body fluids is bound to be faced with many challenges. Sierra Leone experienced two years of a devastating epidemic, the impact of which has been disastrous economically and socially, resulting in significantly higher unemployment, lower schooling and less food consumption [23]. A fragile health system and the challenges related to the EVD response created fear and mistrust in many people [24,25]. This mistrust extended to everything that had to do with “Ebola” and could be clearly observed throughout our study. Moreover, the participants in the SLEVP study had recently survived a deadly disease and many had to deal with its psycho-social and economic consequences. It is well established that EVD survivors are more vulnerable to psychological distress caused by the disease experience as well as by stigmatization and social rejection [26].

### Overall perception of the study

The SLEVP study team, as reflected in our interviews with study staff and study participants, overall showed high professionalism, high work ethics and excellent problem solving capacity, all of which generated an atmosphere of enthusiasm, respect and friendship and contributed to the success of the study. Study participants were especially fond of the respectful atmosphere and the strict confidentiality, study staff mentioned the good quality of the training which prepared them to tackle challenging situations and guided them through the sometimes difficult process of the project. Moreover, the very fact that some of the study staff were already known to the participants and that survivors were employed as liaison officers was an important trust-building measure. The success of these strategies can be seen in the overwhelmingly positive feedback we received from study participants on the study staff, and in the extremely low losses to follow-up [2]. Overall SLEVP study participants conceived the testing of their body fluids positively and saw it as a useful means to know their status and to be able to communicate the results to their partners or to the community, if they wished to. They were also glad for the financial remunerations they received and for the free medical care they were entitled during the study.

Nevertheless, as shown from our results, some study participants were ambivalent and sometimes reluctant towards sampling of certain body fluids (especially semen, blood and vaginal fluid). These reservations could eventually be overcome by guaranteeing confidentiality and privacy, by comprehensive professional counseling and arguments that stressed the benefits for the study participants. Moreover, many of the national SLEVP staff where either themselves survivors or were well acquainted with the clinical treatment of survivors. Still, there seemed to have been a cognitive gap between the signing of the informed consent and the actual sample taking, and between the understanding and perceptions of the study participants and the SLEVP study staff on the study process. To some study participants, the implications of sample taking were not completely clear and it seems that the way the informed consent was explained to the study participants could be improved upon.

The ambivalence and reluctance that were expressed by some of the study participants–the initial rumors that participants would be harmed in the study and that blood would be sold or used during political campaigning–revealed concerns that were present before the actual study process started and when informational meetings with key stakeholders were held. These rumors are indicative of the atmosphere in which the study was started, and, as mentioned above, the mistrust that was felt towards everything that had to do with “Ebola”. On a deeper level, these rumors may have been expressions of broader societal challenges and historical circumstances. They may have revealed uncertainties of who would really profit from the sampling of the body fluids, an issue that is broadly discussed within the scientific community as well. Ethical considerations of biobanking, especially in the context of disease outbreaks and under conditions of socioeconomic inequities, are hotly debated and the ethical adequacy of material transfer questioned [27]. The ownership of samples collected during the West African EVD outbreak is still unclear and an inventory on the location of samples lacking [28].

### The sampling process and gendered dynamics

Ambivalences were expressed throughout the study process, in relation to male and female intimate sampling and to the sampling of blood. Self sampling and masturbation was described by study participants as unusual practices and the latter as prohibited. In the course of the sampling exercise, study participants were obliged to transgress cultural and intimate boundaries, exchanging “indecent” behavior for, in the eyes of the participants, useful health information. Initial consultations with the Islamic council did settle the concerns from the perspective of the study staff but not necessarily for the study participants. Intimate sampling remained deeply concerning for male and female participants alike. Reservations were expressed as fear and anger but also in feelings of loss or becoming sick. Several narratives were related to the loss of semen that would make men weak and powerless, or that the taking of blood would make especially pregnant and elderly women dizzy and sick. Participants were not only worried about the frequency of the blood draw but also of the amount which they often considered “too much”. The study added blood sampling relatively late in the study process and did only draw serum for serological analyses, which may in contrast be less than in other clinical studies. The „removal”narrative clearly goes beyond the simple drawing of a body fluid, creating an illness experience over something being taken away, albeit by consenting. It entangles the deeper meanings and symbolism of „too much blood”and „blood loss”as “blood being stolen”. Similar perceptions are well documented from anthropological research of clinical trials. Saethre and Stadler [29] call them “narratives of harm”. They are “a vehicle through which gender, cash, social reproduction, morality, and medicine were articulated” (p. 104). In their study on negotiating social relations during an HIV trial in South Africa, the authors also describe a similar cognitive gap between the informed consent which in detail explained procedures of taking blood and the feelings of trial participants that the blood taken was excessive and harmful. They contextualize these perceptions within postcolonial relationships of post-apartheid South Africa but clearly, unequal relationships between the global south and the global north, the researched and the researcher, can be observed in many African countries.

While females were depicted by staff as unproblematic and cooperative during intimate sampling, study participants themselves often felt fear, shame and embarrassment. This was overcome in often surprising ways, e.g. in that female staff showed their own nakedness and vulnerability. This gesture is also symbolic of the ambivalence of the study staff who advocated for a western biomedical conception of the naked human body as something natural in a medical encounter, often building a counter-narrative to the female participants who perceived nakedness as being indecent and immoral, the more so when being asked to put a swab into their vagina while menstruating. The discrepancy between staff perceptions of women as being “unproblematic and cooperative” and the women’s reported feelings of fear and shame seem to reflect larger societal gender dynamics. In the study, men may have been more successful to negotiate their position and articulate their needs than women. Even though gender sensitive trainings were part of the study protocol it seems that staff were not able to pick up on these issues in the counseling sessions.

Participants had very creative ways in re-gaining “control” over their body fluids and showing agency. Some male participants were not able or, on rare occasions, not willing to provide intimate sampling on several occasions. This was interpreted by study staff as either post-EVD related impotency or as a form of social resistance. In some instances staff suspected several male study participants to put gel into test tubes. The behavior was said by staff to have caused disturbances during the study process and was seen by some as abusing a system that was built on mutual trust. Still, the actual circumstances seem to tell another story, where missing samples for this reason was uncommon. Again, on a symbolic level very similar accusations are known from Saethre and Stadler [29], when women during the study were requested to apply microbicide gel vaginally. Some who were called “gel-dumpers” were suspected to only pretend the vaginal application in order to receive the remuneration. Gel dumping was considered “irresponsible because it endangered the scientific process” (p. 110).

### Implications for medical studies

It is important to understand the potential importance some of these perceptions have on the recruitment of study participants and the acceptability of studies, first on the symbolic level: Body fluids may be considered as pure or polluted, as powerful substances, inflicting harm or as curative agents. Though not explicitly mentioned by the female participants themselves, female staff confirmed that some of the women considered menstrual blood as “unclean”. Several female participants were especially concerned where their menstrual blood samples would be taken, a worry that was also expressed for other body fluids in male and female participants, such as venous blood and semen. This could indicate a spiritual belief that body substances are generally considered as potentially powerful and able to inflict harm, and used against the person when in the wrong hands. These perceptions are wide-spread in many parts of Africa and have been described e.g. for blood donation in Sub-Saharan Africa [30,31], for blood draw in clinical research [10] and for semen collection in HIV studies [13].

On a structural level, reservations towards biomedical research in low-resourced settings in Sub-Saharan Africa that involves sample taking (usually blood) are not new and have been reported from several studies [10,32,33]. In a study by Newton et al. [12] blood drawn from infants in Ghana led to rumors that it would be used for transfusions for elderly people. These perceptions led to substantial loss to follow up. Similarly, in a study by Nchito et al. [11] in Zambia loss to follow up was partly attributed to the fear of a rumor of blood thefts. Perceptions on body fluids and sample taking can influence decisions of participants to take part in studies and to retain in a study. Yet in the specific cohort study here evaluated, study retention was close to 100%, meaning that especially for some men they remained over a long follow-up time (approaching two years). Factors that have influenced the high retention in the cohort, based on our findings, seem to have been the perceptions of a very high level of trust in the confidential handling of all results, the interest and engagement in knowing ones status as body fluid/semen positive or negative, and the professionalism paired with in-depth community belonging of staff involved. The targeted pre- and post- test counseling offered, as well as the fact that actual sampling of blood was reduced to a minimum, can also have influenced retention positively.

Rumors on medical research or public health interventions in low-income countries can also be seen as an expression of a potentially problematic relationship rooted in history between affected communities and researchers who are often from high-income countries [34–36]. Kovacic et al. [37] for instance found that in Uganda community experience with control programs on sleeping sickness remains in the memories of people for decades and may influence perceptions on medical research today. Feldman-Savelsberg et al. [35] in their research analyzed how colonial history, inter-country political conflicts, insensitive behavior of public health staff and not considering gender issues led to a disastrous chain reaction with rumors and subsequent resistance of girls and their parents towards a vaccination campaign against neonatal tetanus. The question how medical research is perceived in a particular socio-cultural setting has implications for the sustainability of research involving power dynamics between co-researchers and communities under study [34]. However, it is important to neither over- nor underestimate the power of rumors for medical research. While many of our study participants for instance articulated that they did not believe in the rumors, it is nevertheless important to not ignore them as they may pose a threat to medical research [36].

In our evaluation of a cohort study implemented during an EVD emergency, we found that attention to a very high level of confidentiality offered (ID, location of study, staff integrity etc.) as well as the attention to training and involvement of local staff and survivors’ representatives have contributed to the high retention in study and in general positive attitudes of participants. Further we identified challenges such as the need of continued attention to information and consent procedures, where in this study the purposive recruitment in the cohort was facilitated by information meetings and survivors’ liaison officers. Still, some participants felt they had initial doubts of study aims, which were overall resolved during the actual recruitment and consent processes.

It is important to conceive possible concerns towards sampling of body fluids early in the planning phases of a project and have these perspectives broadly discussed. In order to capture any reservations it is necessary to provide plenty of possibilities of information sharing and follow-up of continuous consent.

### Study limitations

This study evaluation was conducted in a post-emergency. For data collection we had to follow a very strict and constrained time format. Interviews and focus group discussions at the two study sites had to take place in parallel, and interviewers sometimes had to conduct 2–3 interviews a day. This rapid study design did not allow for an in-depth ethnographic data collection and we may have missed important information, especially on such sensitive issues the study focused on. However, judging from the sometimes very personal and intimate information we received from our study participants, we got the overall impression that many interviewees were very open and frank when relating their opinions and stories.

Because study participants were invited to the same study site and by the same liaison officers as the viral persistence study, some study participants initially confused our process evaluation with the latter and thought we had come back to do a follow up. These confusions could be resolved by explaining that our study was a separate one and that we wanted to hear their opinion on the viral persistence study. Moreover, we also had different staff employed to do the interviews so as not create any biases regarding confidentiality.

For reasons of practicality, some interviews with female study participants had to be conducted by male interviewers. However, we specifically trained the male interviewers in gender sensitive questioning and RK supervised them closely in the field. All focus group discussions with female participants were conducted by a female interviewer and all focus group discussions with male participants were conducted by a male interviewer.

### Conclusions

In clinical studies that involve the sampling of body fluids it should be part of the program to engage local communities, religious leaders and survivors. It is essential to take into account cultural and religious implications of the collection process and to understand underlying gender dynamics and vulnerabilities. All study staff should be trained in cultural and gender sensitive issues and it should be considered to include social science expertise in all phases of the study process.
